# The development, design, testing, refinement, simulation and application of an evaluation framework for communities of practice and social-professional networks

**DOI:** 10.1186/1472-6963-9-162

**Published:** 2009-09-15

**Authors:** Jeffrey Braithwaite, Johanna I Westbrook, Geetha Ranmuthugala, Frances Cunningham, Jennifer Plumb, Janice Wiley, Dianne Ball, Sue Huckson, Cliff Hughes, Brian Johnston, Joanne Callen, Nerida Creswick, Andrew Georgiou, Luc Betbeder-Matibet, Deborah Debono

**Affiliations:** 1 Centre for Clinical Governance Research, Faculty of Medicine, University of New South Wales, Sydney, NSW 2052, Australia; 2 Australian Institute of Health Innovation, Faculty of Medicine, University of New South Wales, Sydney, NSW 2052, Australia; 3 School of Public Health and Community Medicine, Faculty of Medicine, University of New South Wales, Sydney, NSW 2052, Australia; 4 Health Informatics Research and Evaluation Unit, Faculty of Health Sciences, University of Sydney, Cumberland Campus C42, East St, Lidcombe NSW 2141, Australia; 5 Australian College of Health Service Executives, National Office, Macquarie Hospital, Wicks Road, North Ryde, NSW 2113, Australia; 6 Effective Practice Program, National Institute of Clinical Studies, National Health & Medical Research Council, Level 5, Fawkner Centre, 499 St Kilda Road, Melbourne, Victoria 3004, Australia; 7 Clinical Excellence Commission, Level 3, 65 Martin Place, Sydney NSW 2000, Australia; 8 Australian Council on Healthcare Standards, 5 Macarthur Street, Ultimo NSW 2007, Australia

## Abstract

**Background:**

Communities of practice and social-professional networks are generally considered to enhance workplace experience and enable organizational success. However, despite the remarkable growth in interest in the role of collaborating structures in a range of industries, there is a paucity of empirical research to support this view. Nor is there a convincing model for their systematic evaluation, despite the significant potential benefits in answering the core question: how well do groups of professionals work together and how could they be organised to work together more effectively? This research project will produce a rigorous evaluation methodology and deliver supporting tools for the benefit of researchers, policymakers, practitioners and consumers within the health system and other sectors. Given the prevalence and importance of communities of practice and social networks, and the extent of investments in them, this project represents a scientific innovation of national and international significance.

**Methods and design:**

Working in four conceptual phases the project will employ a combination of qualitative and quantitative methods to develop, design, field-test, refine and finalise an evaluation framework. Once available the framework will be used to evaluate simulated, and then later existing, health care communities of practice and social-professional networks to assess their effectiveness in achieving desired outcomes. Peak stakeholder groups have agreed to involve a wide range of members and participant organisations, and will facilitate access to various policy, managerial and clinical networks.

**Discussion:**

Given its scope and size, the project represents a valuable opportunity to achieve breakthroughs at two levels; firstly, by introducing novel and innovative aims and methods into the social research process and, secondly, through the resulting evaluation framework and tools. We anticipate valuable outcomes in the improved understanding of organisational performance and delivery of care. The project's wider appeal lies in transferring this understanding to other health jurisdictions and to other industries and sectors, both nationally and internationally. This means not merely publishing the results, but contextually interpreting them, and translating them to advance the knowledge base and enable widespread institutional and organisational application.

## Background

Scholars within the health [1], aviation [2], manufacturing [3], finance [4], education [5] and military [6] sectors have argued that collaborating communities and linked, professionalised networks are significant determinants of important outcomes such as work satisfaction [7], motivation [8], recruitment and retention [9], high performance [10], organisational resilience [11], safer organisations [12] and systems renewal [13]. However, there is a paucity of empirical research, as opposed to normative claims in the literature, to support these arguments. Given the substantial resources invested in supporting collaborating structures, the absence of evidence of their effectiveness is a significant problem. This is especially so given the potential benefits of optimizing the performance of large, complex sectors, such as the health system, which is the specific focus of our project.

Communities of practice can be defined as "groups of people who share a concern, a set of problems, or passion about a topic, and who deepen their knowledge and expertise in this area by interacting on an ongoing basis" [14]. Social networks are "set [s] of people ... ['actors'] ... with some pattern of interactions or 'ties' between them ... [eg] friendships among a group of individuals, business relationships between companies" [15]. In one sense they are two sides of the same coin, but communities of practice scholars have tended to emphasise learning processes while social network scholars have focused on underlying structural properties.

These kinds of clustered relationships abound in health care and other industries. They can be naturally-occurring [eg, groups of clinicians who share professional responsibilities, refer patients to each other, jointly manage care and collaborate over complex cases] or mandated [eg, purpose-designed, sponsored, initiated and funded by organisational leaders] [16]. They can also be tangible and geographically-anchored [eg, in one section of an organisation such as a teaching hospital or general practice] or virtual and geographically-dispersed [eg, international colleagues with common interests linked via telecommunications]. Terms for related phenomena include teams [17], sub-cultures [18], micro-systems [19], inter-professional practices [20] and integrated services [21].

Communities of practice [14,22] and social-professional networks [23,24] are increasingly seen as crucial determinants in understanding and enhancing group-oriented services. Most literature supports a position that, although there is much to understand, services to patients [clients, customers, purchasers or consumers in other industries] and workplace cultures can be expected to improve where communities of practice and social-professional networks are emphasised and strengthened, whether these are naturally-occurring and emergent, or mandated and purpose-designed networks [16]. These tend to be theoretical or conceptual claims, however, rather than concerted empirical demonstrations.

At the same time, potentially negative consequences have been noted. Wenger and colleagues argue that communities of practice can encourage the hoarding of information to the detriment of others, curtail improvement and progress, and create in-group and out-group rivalries [14]. Buchanan suggests that networks can be a negative as well as positive force, and can mean all the eggs are in one basket. For instance, if a network [eg, electricity grid, computer system, or clinical collaboration] fails, deleterious consequences can result [23]. Communities of practice and social-professional networks can marginalise others, create factions and silos, become vehicles for elitism or isolationism, strengthen us-and-them behaviours and impede organisational sharing [14,22-31]. Thus we should not accept that communities of practice and social-professional networks are universally useful.

Although interest in communities of practice and social networks in industry settings has grown remarkably, no convincing model for their systematic evaluation has eventuated. Many authoritative commentators agree that analytic evaluation methods and tools are lacking [32-34]. Thus, there is strong consensus amongst scholars that it is vital to develop an appropriate evaluation framework and model with supporting data-gathering tools. If we are to be confident that communities of practice and social networks enable organisational success, and to understand how and to what extent they do so, we need to be able to quantify their contribution to satisfaction, motivation, recruitment and retention, performance, resilience, safety and renewal. Conversely, if these kinds of collaborative structures prove less useful than expected, we need to understand their limitations.

### The case for the project

We reviewed the literature on communities of practice and social-professional networks. Significant researchable characteristics of communities of practice include how learning is enabled and constrained, what can be achieved by actively cultivating them, their relationships to organisational learning, interactivity amongst participants within and between communities of practice, how members join and are socialised, the mechanisms of participation, and the stages of their development [14,22,28-30]. The literature indicates that the core features of social-professional networks are their deep structure, how they enable change, how information is facilitated by and in them, the processes of information distribution and search, the circumstances in which phase transitions occur, connectedness of hubs and nodes and the conditions which dictate their robustness or fragility [23-27]. In our review of social-professional networks, we applied some of these central concepts to key patient safety issues [16]. We concluded that "*Clinicians, like other professionals, work best when they are allowed to flourish in groupings of their own interests and preference, are empowered ... and nurtured and influenced by their peers rather than controlled by others*" [16].

**Figure 1:**
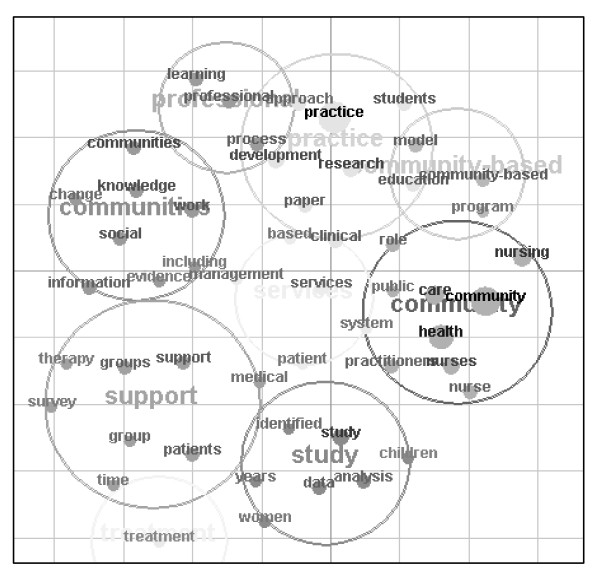
**Map of the content analysis of the health communities of practice literature**.

We also conducted a comprehensive analysis of the communities of practice literature pertaining to the health system [35]. We examined the Medline, CINAHL and Embase databases and performed a snowballing search for additional literature. We identified 624 references of which 90 were research papers. Content analysis of the 624 publications via Leximancer, a data mining tool, allowed us to construct Figure 1 which maps the overarching themes in the literature - the social and professional nature of communities of practice, and their strong relationship to service provision and treatment. There are five major themes in the 90 research papers: organisational change [eg, how communities of practice promote productive change, and how change effects communities of practice]; the impact of technology on communities of practice; the contribution of communities of practice to professional and inter-professional learning and the shaping of organisational knowledge; learning processes within communities of practice, including mechanisms of collaboration, structured reflection and the development of shared competencies; and barriers to participation in communities of practice, particularly mono-professional, organisational, institutional and service boundaries and silos. We concluded our review by synthesising the literature as follows: "*The systematic analysis reveals that the community of practice idea has been empirically applied in the health sector in useful ways, but research is sporadic, and many studies are descriptive and centred on one profession or location. The transferability and generalisability of findings is limited. A difficulty with the empirical studies is ... a lack of specificity in their claims. For example, at times the argument is made that team work has improved but what 'good team work' means is not clarified. There is scope for more far-reaching, rigorous and systemic research" *[35]. This background work leads to the current protocol.

### Aims

This project's specific aims are to develop and design, test in the field, and then refine, simulate and apply a framework, model and tools which can be used to evaluate communities of practice and social networks for their effectiveness and sustainability. We will work in the health system for compelling reasons. As a large, diverse industry (9.3% of GDP)[36] the health system supports many types of policy, managerial and clinical communities of practice and social-professional networks comprising diverse occupational groups, and traverses the public and private sectors. These characteristics and complexities mirror other key industries, greatly enhancing the potential to transfer findings, theories and project outcomes beyond the health sector.

The project thus more broadly aims to realise a scientific innovation of importance. Without an evaluation framework, model and tools, sponsors will continue to be uninformed as to what communities of practice and social networks deliver; will be unable to identify and measure their strengths and weaknesses; and will be frustrated in their efforts to achieve desirable outcomes, and returns on investment. This has profound implications for how communities of practice and social-professional networks are enabled, configured and resourced. Such research is at the heart of a crucial question: how well do groups of professionals work together and how could they be organised to work together more effectively?

The research strategy and process are novel and innovative and are designed to produce original methods in the building of a new framework, model and tools. These include: the engagement of influential stakeholders and consumer groups; theoretical and empirical advancement; a sophisticated new research approach for doing future work; an innovative, tested, refined framework, model and tools for researching and evaluating communities of practice and social-professional networks; and the gathering of original data from the field using the tools to illuminate progress. Our data gathering tools [questionnaires, interview schedules, case study designs, focus group processes, critical incident mapping protocols] will employ new approaches.

We will inform our work using theories on teams [17], inter-professional learning [37], network structures [25,38], change [39] and group dynamics [40]. These are fundamental concepts to our enquiry, guiding our thinking, particularly in the initial and model-building phases. We will, however, mainly inform our project using the empirical literature to rigorously build our research designs on the basis of prior research results. Few research projects are both empirically and theoretically multi-modal in the one study.

## Methods and design

### Conceptual framework

**Figure 2:**
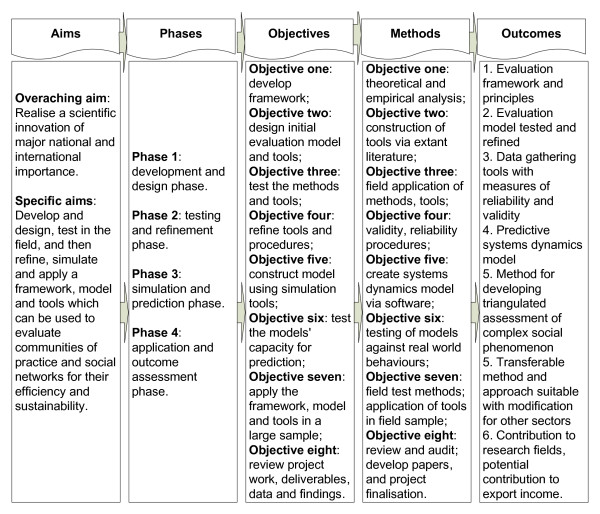
**Conceptual framework, design and methods**.

This is a comprehensive effort to execute a rigorous evaluation methodology, and deliver tools for the benefit of researchers, policymakers, practitioners and consumers. We have the opportunity to achieve both meaningful and transferable advances in the knowledge base of the discipline and to incorporate novel and innovative aims and concepts into the project design. Through the support of key stakeholders within the Australian health system, we will have access to a wide range of workplace communities and networks, including hundreds of clinicians, to test and refine our models. The overarching aims, phases, objectives, methods and outcomes in the approach are depicted in Figure 2. Ethics approval for the study was granted by the University of New South Wales' Human Research Ethics Committee, application number 09085. Informed consent from participants is a key component of the approval.

### Methodology

The methodology is conceptualised in four major phases, matched to the aims and objectives and commensurate with the research approach. Each phase has two components, and involves a little over a year's work to achieve the project's objectives.

#### Phase 1: development and design phase

The project will produce an evaluation framework founded on empirically-grounded evaluation principles. Initially we will drill further into the literature and derive from this assessment a set of fundamental, working evaluation principles. Work feeding into this process includes seminal contributions by Wenger and colleagues [14,22,30] and Watts [41], Strogatz [27] and Barabási [25] as well as our work in health systems social structures and change [42-44].

Secondly, armed with the initial framework, we will design a preliminary evaluation model. There are many evaluation types [45], but our previous research suggests that a triangulated, multi-method model is best suited to this problem. Our earlier work on the evaluation of education processes [44,46], information management [47] and information and communication technologies [48] demonstrates this kind of model can be comprehensive, have high utility and yield robust data. This phase includes the design of data-gathering tools [including questionnaires, interview schedules, case study protocols for video ethnographic work, critical incident maps, and focus group question schedules] to make assessments of communities of practice and social networks, taking into account recent designs [eg, Verburg and Andriessen's] [49]. A key step is the identification of outcome measures to assess the effectiveness of communities of practice and social-professional networks. We have identified indicators for work satisfaction (5 indicators) [50], motivation (6) [51], recruitment and retention (5) [52], high performance (18) [53], organisational resilience (5) [11], safer organisations (7) [16] and systems renewal (11) [54,55], totalling 57 indicators against which metrics are designated.

#### Phase 2: testing and refinement phase

Thirdly, in phase 2, we aim to test the model and tools across a focused sample of communities of practice and social-professional networks. Peak Australian stakeholder groups have agreed to involve a wide range of members and participant organisations in this study. These include networks or communities of clinicians identified by the National Institute of Clinical Studies, accreditation surveyors of the Australian Council on Healthcare Standards, managerial members of the Australian College of Health Service Executives and clinical, managerial and policy groups associated with the Clinical Excellence Commission. We will identify, in conjunction with these stakeholder partners, representative samples of differing types of communities of practices and social-professional networks (naturally-occurring, mandated, geographically-anchored, geographically dispersed) and enrol them in our study. We aim to make comparisons between groups and across group types.

The testing component will yield experience which will, fourthly, enable us to refine the tools, including their validity and reliability. Differing approaches to examine validity and reliability will be taken. Work on the reliability of social network data is important, and optimum outcomes can be achieved by re-applying social network questionnaires and comparing consistency of results [56-58]. We have validated questionnaires and used multi-method approaches to corroborate discrete methods convergently [44,46-48,59].

#### Phase 3: simulation and prediction phase

Phase 3 involves two components: fifthly, simulating the systems dynamics [60] of communities of practice and social networks and sixthly, testing the models' capacity for prediction [61]. Communities of practice and social networks are complex adaptive systems within larger complex adaptive systems. They can be modelled via simulation tools that facilitate the mapping of variables within and external to them. This enables us to understand relationships between parts of the system and to run predictive simulations. Governing features of complex systems include feedback, processes, resources, accumulation of flows into stocks and time delays [62]. In the case of a community of practice of emergency clinicians, for example, key variables include levels and training of staff, workforce casualisation, delays in processes, incentives and disincentives, and factors which enable and constrain behaviours and attitudes. In this phase we will also conduct controlled simulation experiments. We will develop role plays [n = 20] of case scenarios and test in the laboratory the dimensions of role behaviours and decision-making processes under controlled conditions. Theorised barriers and enablers will be tested in idealised circumstances in this way to analyse the scope of the boundaries and assess hypotheses and predictions.

#### Phase 4: application and outcome phase

Having derived the refined evaluation framework, model and tools we will seventhly apply them on a large scale to a sample of communities of practice and social networks identified across Australia, covering the public and private sectors, in order to achieve a sample broadly representative of the health care system. In this phase we will gather sufficient data on communities of practice and social-professional networks to assess their value, and judge the extent to which they contribute to work satisfaction, motivation, recruitment and retention, performance, resilience, safety and renewal. We will test the results and calibrate them against the predictive simulation data drawn from phase 3.

This leads to our eighth component - a review of the research outcomes to interpret results and make final observations on the findings. We will use an arm's length, expert panel to triangulate judgements. Such a process further strengthens project outcomes, closing the feedback loop and helping to determine whether communities of practice and social-professional networks realise the results intended for them.

## Discussion

This protocol provides a conceptual framework and research design for evaluating hard-to-research social science manifestations which are key determinants of health systems performance. A multi-methods, phased research design is important and innovative, and has been matched to the phenomena under investigation. This represents a bold approach to creating and then testing an evaluation model.

### Study strengths and limitations

#### Strengths

Investigating and evaluating complex organisational constructs such as communities of practice and social-professional networks is challenging. Conducting research in naturalistic settings, examining behavioural and attitudinal factors in context, is a key strength. The research design recognises this, and applies a complex systems approach to the examination of complex systems phenomena. Repeated, staged testing of progress with the model introduces validity, reliability and rigour. Incorporating measures of organisational performance in the design increases the potential for robust correlations. Having undertaken extensive literature analyses of both communities of practice and social-professional networks, we have laid a sound platform to aid understanding and provide theoretical and empirical building blocks to our research studies.

#### Limitations

Objective measures are hard to achieve in social science research, as is generalisability, thus we have incorporated triangulation techniques in our design and seek transferability of findings rather than strict generalisability. Nevertheless, our results by definition will fall short of the gold standard, and will not produce level 1 evidence. Balancing this limitation is the reality that a strict test of this kind cannot be applied against research in this mould. Thus, other safeguards, including seeking data from multiple sources and assessing the extent to which they converge or diverge, constitute an effective, alternative approach.

## Conclusion

This is a large, comprehensive research project which will deliver outcomes both specifically relevant to health systems and transferable to the large number of organisations, systems and sectors which are affected by the effectiveness, or otherwise, of communities of practice and social-professional networks. In developing a rigorous evaluation methodology and delivering tools for researchers, policymakers, practitioners and consumers, the research project also demonstrates innovation and tests novel approaches in design. By interpreting and translating results for applications both within and beyond the health system, the project will advance understanding of how professionals work together, and how to optimise this interaction to improve organisational effectiveness and service delivery.

## Competing interests

The authors declare that they have no competing interests.

## Authors' contributions

JB and JIW designed the study, phases, protocol and instruments, and wrote the manuscript. GR, FC, JP and JW are members of the research team, and commented on an earlier draft of the manuscript. DB, SH, CH and BJ are industry partners, are members of the expert advisory committee, and provided input into the initial conceptualisation of the project. JC, NC, AG and LB-M commented on an earlier draft of the manuscript, and are members of the expert advisory committee. DD commented on an earlier draft of the manuscript and developed the ethics application, and steered it through the approval process. All authors approved the final version of the manuscript.

## Pre-publication history

The pre-publication history for this paper can be accessed here:



## References

[B1] Kvarnström S, Cedersund E (2006). Discursive patterns in multiprofessional healthcare teams. Journal of Advanced Nursing.

[B2] Stout R, Salas E (1997). The role of trainee knowledge structures in aviation team environments. International Journal of Aviation Psychology.

[B3] Coupland C, Blyton P, Bacon N (2005). A longitudinal study of the influence of shop floor work teams on expressions of 'us' and 'them'. Human Relations.

[B4] McKee T (2003). Building successful teams in the midst of transition. The Journal for Quality and Participation.

[B5] Bleakley A (2006). Broadening conceptions of learning in medical education: the message from teamworking. Medical Education.

[B6] Wright MC, Kaber DB (2005). Effects of automation of information-processing functions on teamwork. Human Factors.

[B7] Rosenfeld LB, Richman JM, May SK (2004). Information adequacy, job satisfaction and organizational culture in a dispersed-network organization. Journal of Applied Communication Research.

[B8] Ardichvili A, Page V, Wentling T (2003). Motivation and barriers to participation in virtual knowledge-sharing communities of practice. Journal of Knowledge Management.

[B9] Haesli A, Boxall P (2005). When knowledge management meets HR strategy: an exploration of personalization-retention and codification-recruitment configurations. The International Journal of Human Resource Management.

[B10] Pathirage CP, Amaratunga DG, Haigh RP (2007). Tacit knowledge and organisational performance: construction industry perspective. Journal of Knowledge Management.

[B11] Weick K, Sutcliffe K (2007). Managing the unexpected: resilient performance in an age of uncertainty.

[B12] Weingart SN, Page D (2004). Implications for practice: challenges for healthcare leaders in fostering patient safety. Quality & Safety in Health Care.

[B13] Antonacopoulou E, Ferdinand J, Graca M, Easterby-Smith M (2005). Dynamic capabilities and organizational learning: socio-political tensions in organizational renewal.

[B14] Wenger E, McDermott R, Snyder W (2002). Cultivating communities of practice.

[B15] Newman MEJ, Watts DJ, Strogatz SH (2002). Random graph models of social networks. Proceedings of the National Academy of Sciences of the United States of America.

[B16] Braithwaite J, Runciman WB, Merry A (2009). Towards safer, better healthcare: harnessing the natural properties of complex sociotechnical systems. Qual Saf Health Care.

[B17] Gosling AS, Westbrook JI, Braithwaite J (2003). Clinical team functioning and IT innovation: a study of the diffusion of a point-of-care online evidence system. Journal of the American Medical Informatics Association.

[B18] Braithwaite J, Westbrook MT, Iedema R, Mallock NA, Forsyth R, Zhang K (2005). A tale of two hospitals: assessing cultural landscapes and compositions. Social Science & Medicine.

[B19] Mohr JJ, Batalden PB (2002). Improving safety on the front lines: the role of clinical microsystems. Quality & Safety in Health Care.

[B20] Braithwaite J, Travaglia J (2005). The ACT Health inter-professional learning and clinical education project: discussion paper #2 [inter-professional practice].

[B21] Ballard DJ (2003). Indicators to improve clinical quality across an integrated health care system. International Journal of Quality in Health Care.

[B22] Wenger E (1998). Communities of practice: learning, meaning and identity.

[B23] Buchanan M (2003). Nexus: Small worlds and the groundbreaking science of networks.

[B24] Watts DJ (2004). Six degrees: The science of a connected age.

[B25] Barabási A-L (2002). Linked: The new science of networks.

[B26] Johnson S (2001). Emergence: the connected lives of ants, brains, cities and software.

[B27] Strogatz SH (2001). Exploring complex networks. Nature.

[B28] Boland R, Tenkasi R (1995). Perspective making and perspective taking in communities of knowing. Organization Science.

[B29] Honeyman A (2002). Communities of practice. British Journal of General Practice.

[B30] Lave J, Wenger E (1991). Situated learning: Legitimate Peripheral Participation.

[B31] Cott C (1997). 'We decide, you carry it out': A social network analysis of multidisciplinary long-term care teams. Social Science and Medicine.

[B32] Lesser EL, Storck J (2001). Communities of practice and organizational performance. IBM Systems Journal.

[B33] Dubé L, Bourhis A, Jacob R (2006). Towards a typology of virtual communities of practice. Interdisciplinary Journal of Information, Knowledge, and Management.

[B34] Mattick JS, Gagen MJ (2005). Accelerating networks. Science.

[B35] Greenfield D, Travaglia J, Nugus P, Braithwaite J (2007). A review of health sector community of practice literature: final report.

[B36] Australian Institute of Health and Welfare (2008). Australia's health. Canberra: AIHW.

[B37] Braithwaite J, Travaglia J (2005). Inter-professional learning and clinical education: an overview of the literature.

[B38] Edwards N (2002). Clinical networks. British Medical Journal.

[B39] Braithwaite J (1995). Organisational change, patient-focused care: an Australian perspective. Health Services Management Research.

[B40] Levi D (2007). Group dynamics for teams.

[B41] Watts DJ, Strogatz SH (1998). Collective dynamics of 'small-world' networks. Nature.

[B42] Braithwaite J (2006). Analysing social structural and cultural change in acute settings using a Giddens-Weick paradigmatic approach. Health Care Analysis.

[B43] Braithwaite J (2006). An empirical assessment of social structural and cultural change in acute settings. Health Care Analysis.

[B44] Braithwaite J, Westbrook M, Travaglia J, Iedema R, Mallock N, Long D, Nugus P, Forsyth R, Jorm C, Pawsey M (2007). Are health systems changing in support of patient safety? A multi-methods evaluation of education, attitudes and practice. International Journal of Health Care Quality Assurance.

[B45] Øvretveit J (2002). Action evaluation of health programmes and changes: a handbook for a user-focused approach.

[B46] Westbrook M, Braithwaite J, Travaglia J, Long D, Jorm C, Iedema R (2007). Promoting safety: varied reactions of doctors, nurses and allied health professionals to a safety improvement program. International Journal of Health Care Quality Assurance.

[B47] Braithwaite J, Travaglia J, Westbrook MT, Jorm C, Hunter C, Carroll K, Iedema R, Ekambareshwar M (2006). Evaluation of the Incident Information Management System in New South Wales - overview report.

[B48] Westbrook JI, Braithwaite J, Georgiou A, Ampt A, Creswick N, Coiera E, Iedema R (2007). Multi-method evaluation of information and communication technologies in health in the context of wicked problems and socio-technical theory. Journal of the American Medical Informatics Association.

[B49] Verburg RM, Andriessen JHE (2006). The assessment of communities of practice. Knowledge and Process Management.

[B50] Faragher EB, Cass M, Cooper CL (2005). The relationship between job satisfaction and health: a meta-analysis. Occupational and Environmental Medicine.

[B51] Denier E (2006). Guidelines for national indicators of subjective well-being and ill-being. Applied Research in Quality of Life.

[B52] Holton JA (2005). Rehumanising knowledge work through fluctuating support networks: a grounded theory. The 37th World Congress of the International Institute of Sociology: 2005; Stockholm, Sweden.

[B53] Roberts J (2006). Limits to communities of practice. Journal of Management Studies.

[B54] Watson J, Ovseiko P, eds (2005). Health care systems: major themes in health and social welfare.

[B55] Boon H, Verhoef M, O'Hara D, Findlay B (2004). From parallel practice to integrative health care: a conceptual framework. BMC Health Services Research.

[B56] Creswick N, Westbrook J (2006). Examining the socialising and problem-solving networks of clinicians on a hospital ward. Conference Proceedings of Social Science Methodology Conference of the Australian Consortium for Social and Political Research (ACSPR).

[B57] Creswick N, Westbrook J (2007). The medication advice-seeking network of staff in an Australian Hospital renal ward. Third International Conference on Information Technology in Health Care: Socio-technical Approaches: 2007; Amsterdam.

[B58] Creswick N, Westbrook J Social network analysis of medication advice-seeking interactions among staff in an Australian hospital. International Journal of Medical Informatics.

[B59] Braithwaite J, Hu W, Sorensen R, Patterson R, Meyerkort S, Salkeld G, Zhang K, Mallock N, Iedema R, Betbeder-Matibet L (2002). Report: Evaluation of the Clinical Practice Improvement Training Program.

[B60] Cook R, Rasmussen J (2005). "Going solid": a model of systems dynamics and consequences for patient safety. Quality & Safety in Health Care.

[B61] Lattimer V, Brailsford S, Turnbull J, Tarnaras P, Smith H, George S, Gerard K, Maslin-Prothero S (2004). Reviewing emergency care systems I: insights from systems dynamics modelling. Emergency Medicine Journal.

[B62] Sterman JD (2000). Business dynamics: systems thinking and modeling for a complex world.

